# Capability Approach and Inclusion: Developing a Context Sensitive Design for Biobased Value Chains

**DOI:** 10.1007/s10806-023-09901-1

**Published:** 2023-02-17

**Authors:** Lotte Asveld, Zoë Houda Robaey, Sara Francke, Patricia Osseweijer

**Affiliations:** 1 Department of Biotechnology, Delft University of Technology, Delft, Netherlands; 2 Department of Social Sciences, Wageningen University, Wageningen, Netherlands; 3 DSM, Baden-Würtemberg, Duitsland, Germany

**Keywords:** Inclusion, Capability approach, Biobased value chains, Context sensitive design

## Abstract

Biomass such as crops and agricultural waste is increasingly used as the primary resource for products like bioplastics and biofuels. Incorporating the needs, knowledge, skills and values of biomass producers in the design of global value chains – the steps involved in creating any finished product from design to delivery – can contribute to sustainability, reliability and fairness. However, how to involve biomass producers, especially if they are resource poor, remains a challenge. To make sure that inclusion in global biobased value chains is both fair and effective, the capabilities of relevant actors need to be taken into account, especially of those producing biomass. Access to resources determines to what extent a specific actor can participate in a global value chain. Therefore, differences in capabilities should be a central consideration when new (biobased) value chains are designed. Using the capability approach as an ethical framework to realize inclusion, we discern three complementary strategies for setting up inclusive value chains. Firstly, designing for local conversion factors second, providing adaptive design for new capabilities, and third, investing in local conversion factors. Applying these strategies can lead to context-sensitive design of biorefineries that allow for true inclusion of local stakeholders. We support these claims with reference to case-studies of sugarcane production in Jamaica, modified tobacco in South Africa and the non-edible parts of corn (stover) in the US.

## Introduction

Biomass such as crops and agricultural waste is increasingly used as the primary resource for products like bioplastics and biofuels, which provide an alternative to fossil-based energy and materials. Biobased applications hold the promise of slowing climate change and reducing dependency on fossil resources, while at the same time providing an incentive for socio-economic development and sustainable agriculture (Kline et al., 2017).

Biobased value chains typically build on biomass derived from a biomass-rich region, from where it is exported to a technology-intensive region to be processed into a chemical product, for example, base chemicals or fuels, or products such as plastics or biobased lubricants. However, there are many uncertainties about how to organize biobased value chains in a sustainable and economically fair way (Asveld & Stemerding, 2017), especially because these value chains usually have a global set-up, while the production of biomass takes place in widely diverging local contexts (Meckenstock et al., 2016).

Involving local stakeholders in the set-up or design of biobased value chains can be a way of dealing with the many uncertainties surrounding the set-up of global biobased value chains (Rist et al., 2007); local producers often have valuable knowledge about land management, their natural environment and the associated biomass (Sumane et al., 2018). Such knowledge is important in understanding how to design biobased value chains that are sustainable (Parada et al., 2021). Involving local biomass producers can thus be expected to have both epistemic and moral benefits. While inclusion can lead to more sustainable value chains, it can also bring about distributive justice, an issue not always well addressed in this context (Asveld et al., 2019).

However, biomass producers are often located in the Global South where conditions for inclusion can be suboptimal, for instance because of weak institutions (Postal et al., 2020)^1^. Additionally, cultural values and practices between producers and other actors in agricultural (and biobased) value chains may vary widely (Ros-Tonen et al., 2019; German et al., 2020) as may access to resources (Devaux et al., 2018). Historically, these differences have not always been well addressed in the setting up of biobased value chains and this has contributed to their failure (Romijn & Caniëls, 2011) or created new economic dependencies making local biomass producers more vulnerable (Bottazzi et al., 2018).

In this paper we want to address the question of how to achieve meaningful inclusion in global biobased value chains?

We conducted three case-studies in diverging contexts, as part of a study on inclusive biobased innovations. In this study we engaged with several actors along existing or developing global biobased value chains to find out how such value chains could be set up in an inclusive manner to support both epistemic, moral and instrumental goals.

We argue that for meaningful inclusion, opportunity for local actors should be central to the set-up of the value chain. Inclusion should revolve around increasing opportunities for those least well off (Bryden et al., 2017). Increased opportunities not only provide individuals with the option to shape their lives as they see fit, by reducing stifling economic dependencies, but they also allow for diversity and human flourishing, which is what inclusion should be about. At the same time, the value chain should be designed in alignment with existing local capacities and resources, such as skills and knowledge, to increase its chance of operating effectively and successfully.

We see the Capability Approach as a useful framework for identifying the distribution of both opportunities and actors’ capacities across a value chain, since the Capability Approach has freedoms or opportunities at its core (Sen, 1990; Robeyns, 2017). It differs from many other approaches to developing inclusive agricultural value chains as these often focus on improving economic and labour conditions. These approaches fail to provide a holistic approach to the challenge of ensuring that resource-poor farmers benefit from value chains (Devaux et al., 2018). Applying the Capability Approach to the design of inclusive (biobased) value chains can address these challenges, because it takes the resources of small-scale producers as a starting point for developing the value chain. This approach will also consider technological aspects, pivotal for inclusion in biobased value chains which are fundamentally shaped by emerging technologies. Linking the design and selection of technologies to the capabilities of local actors presents a novel layer to the development of inclusive global value chains.

We will describe three complementary strategies for achieving inclusive biobased value chains. They accommodate existing local capacities as well as create new opportunities, while considering the technological contexts of these value chains. We will illustrate our approach with a case-study in Jamaica, a case-study in South Africa, and a case-study in Iowa, US.

## Inclusion

### Inclusion in Biobased Value Chains

Although the need for inclusive biobased value chains may be clear, what is exactly implied by inclusion also needs to be defined. We conceptualize inclusion in value chains as interventions focussed on those least well off that aim to increase their opportunity to lead a life worth living, without harming those opportunities for other actors in the value chain. Actually, we assume that building inclusive biobased value chains will benefit *everyone* participating in that value chain, for reasons mentioned above.

Inclusion should be integrated in the processes supporting the building of these value chains. These processes encompass the invention, design, development and distribution of the benefits of biobased products (Heeks et al., 2014). This emphasis on inclusion in processes is crucial for value chains since local actors do not participate in value chains as consumers, but as producers of feedstock. This implies that inclusion should not be limited to improving the socio-economic conditions of local stakeholders through dedicated products, but that the preferences, values and skills of local stakeholders should be taken into account in development and decisions on processes supporting the value chain (Chamberlain & Anseeuw, 2019).

Therefore, any approach to inclusion in biobased value chains needs to account for differences in skills and knowledge that individuals have, as well as economic, social and institutional inequalities, and the wide-ranging variety in cultural contexts and associated norms that form the backdrop of global value chains and shape the ‘opportunity structure’ of individuals (Alsop & Heinsohn, 2005). The concept of capabilities is useful here because it helps to identify the distribution of opportunities among the various actors. These can be seen as more structural elements of inclusion than other relatively confined indicators such as increased income or improved sanitation (Oxoby, 2009).

We have not focused on procedural justice here, because the conditions for participation and just procedures can be suboptimal in some contexts, for instance because of lack of trust in authorities or political inequality (Postal et al., 2020). Then, other factors such as institutional arrangements and design choices can be more important than participation for achieving inclusion (Robaey et al., 2022).

### Capabilities and Inclusion

The values of distributive justice and agency that underly the goal of inclusion, are also central to the capability approach. It is more valuable to choose for yourself which kind of life is best for you, rather than having someone else determine your wellbeing (Sen, 1985). Also, acknowledging human diversity is a key feature of this approach and it has often been applied in non-Western contexts (Robeyns, 2017).

The capability approach attaches central importance to individual human capabilities, or ‘a person’s real *freedoms* or *opportunities*’ (Robeyns, 2017) to do and be what (s)he has reason to value. Examples are the capability of becoming educated, of living a healthy life and of growing one’s own food (Nussbaum, 2000). Whether such capabilities are actually available to an individual depends on a range of interconnected inputs, such as institutions, public goods, social practices, resources and skills, also known as ‘conversion factors’ (Robeyns, 2005). When people exercise their agency, they make choices about which of their capabilities to turn into so-called ‘functionings’ (states or activities that create wellbeing (e.g., actually getting educated or living a healthy life). So, capabilities represent options, not outcomes.

The capability approach or framework should be considered a flexible approach that can be adapted to its specific use (Papioannou, 2014). Capabilities can be used to identify relevant social metrics for quantitative studies (see for instance the UN Human Development Index), for ‘thick’ empirical qualitative analyses or for conceptual analysis. Different tools from the capability toolbox can be applied for different purposes (Robeyns, 2017), such as evaluating the appropriateness of technological innovations to a specific context (Oosterlaken, 2015).

In the context of technology development (and many other contexts), the concept of ‘conversion’ of resources is essential. ‘Conversion factors’ refer to factors that allow or hinder one from turning an actual resource into a capability. These factors can be either personal, social or environmental (Robeyns, 2017). Following Haenssgen & Ariana (2018), we include the category of technological conversion factors here. Oosterlaken (2009) gives the example of someone with paralyzed legs who is unable, due to this personal physical characteristic (a personal conversion factor), to convert the resource of a bike into the capability of transporting themself. Instead, such a person would need an adapted bike (a technological conversion factor) that can be operated by hand, for instance. In this case, adapting the design of the technology has delivered the specific capability of transporting themself available to the person with paralyzed legs. Of course, other conversion factors are also still relevant. The adapted design only allows this person the capability of transport if the roads are in good shape (environmental conversion factor) and there is social support for disabled people to move around independently (social conversion factor).

Considering technological conversion factors for achieving inclusion in value chains raises a specific dilemma. As said above, it makes sense to design technologies in alignment with existing capabilities, such as for instance designing a hand bike for a person with paralyzed legs. However, when we see inclusion as creating opportunities, we may expect individuals to increase their capabilities *because of* conversion factors such as technologies. Should we, then, design for existing capabilities, such as present level of education, meaning we use relatively simple technologies? Or should we design for future, desirable capabilities, such as access to a higher level of education and hence more sophisticated technological skills, capabilities that may have come along through increased income because of inclusion in a global value chain? While the hand bike clearly creates new opportunities for a person with paralyzed legs, the choice of technology in biobased value chains can create a different set of new opportunities, either adapted to existing skills and capacities, or oriented to future development.

### Capabilities in Biobased Value Chains

We argue that a diversity of approaches can exist alongside each other. We see three types of overlapping and complementary strategies that can be employed by actors setting up a commercial inclusive biobased value chain: design for existing conversion factors, provide adaptive design for new capabilities and invest in new conversion factors. We derived these three strategies from the case-studies. Combining these strategies can be a way of accommodating both existing *and* evolving capabilities.

In these specific cases two capabilities emerged as most relevant and hence these are the focus of our analysis. Firstly, the capability of reaching financial security is a prominent capability that can be created or enhanced through biobased value chains. This is partly because the specific value chains we studied are commercial enterprises, operated by companies. Although companies can sometimes take responsibility for public issues that go beyond profitability, such as sustainability and access to education (Scherer & Palazzo, 2011), in a global, culturally diverse setting it can be complicated and even counterproductive for foreign companies to invest in local public goods. Additionally, a secure income is also something the local producers of biomass mentioned frequently as of great importance to them, in all three cases.

We assume that the capability of having financial security can be a solid basis for developing other capabilities, such as access to healthy food and the possibility of transporting oneself. However, we do not claim that the capability of having financial security should always be the main goal of any value chain, as other capabilities can also be realized though the inclusive design of value chains such as the capability of having equal opportunity regardless of gender, the capability of enjoying nature or the capability of bodily health, to name just a few. A focus on these capabilities would most likely have required the involvement of governmental actors or a civil society organization, neither of which were a partner in our project.

There is a second capability that is essential to our analysis, which is the capability of controlling one’s environment. This capability underlies the effort of inclusion as this capability enables one to shape life to one’s own desires. Also, the need to exercise control over one’s life, to make autonomous choices, was often mentioned by local producers of biomass, although exactly what this amounted to differed between contexts, as will be discussed below. In the context of biobased value chains this capability can also be framed as the capability of participating in decision making about the design of the biobased value chain. Although these two capabilities are central to our current analysis, we argue that the strategies we develop through these case-studies can also be applied when the focus is on other capabilities.

## Three Cases

To explicate how the concepts of capabilities might be applied to biobased value chains, we refer to three case-studies, one from Jamaica, one from South Africa and one from the United States. All case-studies are part of a research project called IBIS: Inclusive biobased innovation: Securing sustainability and supply through farmers’ involvement. We visited Jamaica in January 2018, the United States in October and November 2018 and again in January 2019, while we conducted remote interviews in the case of South Africa over the course of 2020, supplemented by interviews carried out by local research assistants.^2^

For the Jamaica case-study, we interviewed 15 people, including representatives from the Sugar Industry Authority, the Jamaica Cane Farmers Association and from local sugar cane factories. We also attended meetings of Cane Farmers. For the Iowa case-study, we interviewed 10 people and held two workshops with farmers and representatives of the companies DSM and Poet and local academics. For the South Africa case-study, we interviewed about 25 people, some in groups. For this case-study we interviewed farmers, farm workers, representatives from Bafokeng Nation, SunChem and academics with relevant knowledge of the case.

For the purpose of this paper, we will mostly rely on a rather general description of the cases, because they serve mainly to bring out the challenge in building inclusive value chains and shed light on how the capability approach can contribute to this effort. The description of the cases below is, to a large extent, the result of those interviews and our own field study observations. 3

These three cases are interesting to compare because they involve local actors with very different capability sets. The capabilities of the sugar cane farmers in Jamaica are limited as compared to the capabilities of the corn farmers in Iowa. The Royal Bafokeng Nation in South Africa is a community whose capability set can be located somewhere in between those two. This difference was especially visible in relation to the capability of participating in setting up the value chain, as will be explained later.

Additionally, the cases present different stages of maturity of global biobased value chains. In each of these different stages, other types of value chain design activities turn out to be relevant. The value chain in Jamaica is still in an early conceptual phase, which means that the design space is still very open, i.e., many choices remain to be made (cf. Palmeros Parada et al., 2018). The value chain in South Africa is in the first phases of implementation: relevant actors have been identified and are engaged, and first trials have been executed, so first fine-tuning and adaptations have been made to the original design. The value chain in Iowa has 10 years of experience as the largest second-generation biorefinery in the world, and currently uses this experience to advise other similar projects. This variety in maturity sheds light on the different ways in which inclusion can be realized at different stages of the value chains. We do not identify all three strategies mentioned above in each of the three cases, but rather show how they may be applied in different contexts, and which one of them is most suitable for a specific context.

### Sugar Cane in Jamaica

Jamaica traditionally has a large sugar cane industry. It is one of the main crops grown on the island and many people are dependent on it, although production and export have consistently dwindled since the 1960s (Andrieu, 2022). Many sugar cane farmers are struggling because they own only a very small piece of land. The farming community is aging rapidly and has little access to funds to invest in new technologies or even in new plants. Farming is heavily dependent on government funding (Stanberry, 2022), while at the same the farmers distrust the government.

The community is desperate for new opportunities (Andrieu, 2022). A lot of sugar is imported, because it is cheaper and of more reliable quality (Burrell, 2016, Courtland, 2017). The infrastructure is unreliable. The roads are not well looked after. Climate change is posing many challenges. Harvests are failing because the weather conditions change very quickly and the climate has become too humid to give a good sugar cane yield.

Within this context, the Jamaican Sugar Authority along with co-operatives of sugar cane farmers were looking for a way to diversify their income. This caught the attention of some commercial actors looking for new opportunities in the bioeconomy, although none of them had very concrete plans. However, building on the intentions of these actors and local conditions we made a conceptual design of a biorefinery (Francke, 2019). From this we derived recommendations for designing for existing conversion factors.

#### Designing for Existing Local Conversion Factors

Many Jamaican farmers are desperate to diversify their income. At the time of our visit, they were considering selling sugar cane juices. Selling sugar cane to a biobased value chain, for instance to produce biofuels, may be an option for them. However, at present it is difficult for local sugar cane farmers to achieve the capability of securing financial stability through a biobased value chain. At the time we visited Jamaica, no actual biobased value chain had been set up yet, so in this case the considerations of inclusion mainly concern the concept design.

Many of conversion factors needed for this capability are not available. For instance, the bad condition of the roads means that transporting large quantities of biomass is problematic (environmental conversion factor). Additionally, the state-of-the-art technology that would allow the processing of large quantities of biomass would require highly skilled employees who are not locally available (social conversion factor). Training farmers to grow a different crop, like King’s grass which resembles sugar cane, can be problematic as farmers in Jamaica are reluctant to change and hesitate to adopt new practices. This is partly fuelled by a distrust of new ‘white’ or Western knowledge, a distrust that stems from a history of slavery and discrimination, and partly because of a historical attachment to sugar cane (see also Adams, 2015). So, although they may have the capability of growing a different, more efficient crop, the social custom of staying with sugar cane as a main crop prevents them from turning this into an actual functioning (social conversion factor).

How can the capability of having financial security and the existing conversion factors be taken into account when designing the actual value chain? First, a choice can be made to stay with sugar cane as the main feedstock, even if this might not give the highest yield. Such a choice allows the local farmers to extend their current sugar cane practices without having to adopt new practices, thereby respecting prevalent social norms.

Additionally, ethanol production from sugar cane is well known worldwide^4^. Choosing to implement ethanol as the main product would allow actors working in the Jamaican sugar industry to copy and implement practices that are already in place elsewhere. This fits with the relatively conservative attitude of many relevant actors in the very long-standing sugar cane industry established in colonial times (personal conversion factor). Therefore, building a biobased value chain on sugar cane and well-known processing technologies for ethanol can provide effective technological conversion factors for diversifying income for local farmers.

Ethanol production processes could be complemented by advanced bagasse-to-energy technologies. (Bagasse is a waste product from sugar cane processing.) Relevant actors in the Jamaican sugar industry are already well acquainted with technologies that convert this bagasse to energy (social conversion factor), although the presently used machinery has limited capacity and is outdated (technological conversion factor). The installation of modern high efficiency steam turbines and steam driven mills could prove a valuable asset to the economic viability and sustainability of the Jamaican sugar industry. The feedstock, the technology and the market (integrated use in factories or delivering to the local grid) are all readily available (environmental conversion factors). Applying advanced bagasse-to-energy technologies is promising in turning the capability of sustainable energy production into a functioning.

#### Adaptive Design for New Capabilities

However, applying more advanced technologies such as on-site enzyme production for ethanol fermentation, on-site wastewater treatment and the production of chemical building blocks from biobased resources should be considered as beyond the scope of a Jamaican sugar-based value chain, for now. Given the local available skills, knowledge and learning culture (social and personal conversion factors), as well as the business climate in which access to financial resources are limited (environmental conversion factor), it is unlikely that such advanced technologies would be implemented successfully at this stage.

However, if the biobased value chain proves successful, it will provide local actors with more capabilities, such as access to funds for investments or higher levels of education. This could lead to beneficial conditions for more high-tech applications. The advanced technologies mentioned could be added to the originally proposed biorefinery. Such adaptability appears to be a specific feature of biorefineries, consisting of a collection of technologies where simple first-generation production units can be extended to optimize energy use and product diversity towards more complex second-generation factories. This makes biorefineries especially suitable for adaptive design for new capabilities.

### Solaris in South Africa

The case-study in South Africa concerns the partnership led by the Italy-based company Sunchem, under the name ‘Reya Fofa’ (‘we are flying’), in 2018. Working together with, among others, the Royal Bafokeng Nation (RBN), a value chain would be set up to produce local biobased feedstock using the Solaris crop. This is a nicotine-free, non-GMO tobacco crop. All parts of the plants of the Solaris crop can be used: seeds and leaves for oil, and the leftovers (‘cake’) for the production of feedstock for animals used in South African Airline’s catering. The partnership expected to deliver up to 20% of the diesel used for ground handling services at the O.R. Tambo International Airport by 2023 (Reya Fofa 2019).

The RBN is a community in the northeast of South Africa (the Rustenburg area). As a relatively wealthy and independent part of South Africa, The RBN is considered a unique community on the African continent (Cook, 2013; Manson & Mbenga, 2003). For the RBN community, the Solaris project would mean job creation and improved livelihoods (Reya Fofa 2019). Although mining of platinum and chrome are important sources of income and employment for the RBN community, these are considered unsustainable in the long term and unemployment rates are high (cf. Flomenhoft 2019). The collaboration with Sunchem would be part of RBN’s vision to accelerate socio-economic development in the area.

#### Designing for Existing Local Conversion Factors

The Solaris crop presents a good example of what biobased design for capabilities might look like. The idea behind this crop is that the decline of the tobacco market will leave many tobacco farmers without an income, removing the capability of leading a financially secure life. Solaris would offer these farmers an alternative source of income aligned with their existing knowledge and practices (personal conversion factor).

Additionally, Solaris can be used for multiple purposes. The leaves can be used for fodder while the oil that can be squeezed from the leaves has a wide range of industrial applications. Solaris thereby offers the farmers an *opportunity* to take part in a variety of value chains, with the choice of which market to enter left up to the farmers themselves. However, Solaris also has its limits in terms of design for capabilities. As it turns out, tobacco farmers are not necessarily looking for alternative applications for tobacco, not in South Africa in any case, where Sunchem was looking to sell Solaris. The original tobacco market still offered sufficient options for financial security.

#### Investing in New Conversion Factors

Sunchem’s eventual partner, RBN, did not have any experience in growing tobacco, and only limited experience with farming. RBN derives its income mainly from mining platinum. However, the community foresees that the mines will be depleted at some point, and knows that it needs alternative sources of income (Zvarivadza, 2018). Growing Solaris on the unused land on top of the mines presented an interesting alternative.

To make this viable, Sunchem set up sites and paid RNB farmers to experiment with the crops. Sunchem also employed a learning officer to help with this, as did RBN. By doing this, they stimulated co-learning, an important step in the possible adoption of a novel crop such as Solaris. Both parties were therefore invested in the social conversion factor of active learning practices, thereby enhancing local RBN members’ capability of securing income. Additionally, Sunchem made an effort to build up a local value chain around the Solaris crop, by engaging with possible customers. In this way, they gained the interest of, for instance, Swissport, a company taking care of on-ground transport at Tambo International Airport. Sunchem therefore invested in establishing favourable economic conditions for a Solaris value chain (social conversion factor).

Unfortunately, this project was seriously undermined by the Covid-19 pandemic. It was impossible for Sunchem to keep developing the value chain remotely and many of the potential customers, such as airlines looking for sustainable biofuels, suffered big economic losses. Overall, due to the pandemic, the conditions for building innovative biobased value chains were pretty suboptimal and the project is on hold, for now.

### Corn Stover in Iowa, USA

The case-study in the state of Iowa, USA concerns the Poet/DSM plant for the second-generation bio-ethanol Project Liberty. Iowa has approximately 86,900 farms, more than 97% of them owned by farm families. In 2019, Iowa farmers produced around 2.58 billion bushels of corn for grain and harvested 13.1 million acres (Iowacorn, 2021).

While most of the corn in the region is produced for feed or first-generation ethanol (Bain & Selfa, 2013), the fact that there is no livestock industry nearby means that bales of stover, -the leaves, stocks and cobs left over after harvest-, which are perishable and difficult to transport, do not have an immediate market. The DSM/Poet biorefinery is crucial to the new value chain for corn stover for bio-ethanol production, as it is the only buyer.

With ten years of experience in setting up the world’s largest second-generation bio-ethanol production, our partners at Poet/DSM have sought to continue improving farmers’ involvement and despite no longer running the second-generation biorefinery, still provide advice on setting up similar projects.

#### Designing for Existing Local Conversion Factors

The new second-generation ethanol plant presents another example of designing for existing conversion factors, as it extended local farmers’ capability of having financial security through selling excess corn stover. In doing so, the project relied on existing infrastructure for collecting corn (environmental conversion factor) and the sustainability promise of producing ethanol from lignin. This promise became reality through advanced enzymology developed by DSM (technological conversion factor). However, the Iowa case-study also illustrates an interesting dilemma for designing for capabilities since not all farmers were willing to deliver their corn stover to the Poet/DSM plant and some had to be persuaded before they obliged.

The corn farmers in Iowa have a wide-ranging capability set due to the supporting conversion factors that they enjoy. They have access to financial and technological resources (Bain & Selfa, 2013), they have access to reasonably good infrastructure and education (Zhang et al., 2018). However, people in Iowa welcome diversification of income since unemployment is on the rise and many young people are unable to find the capital investment to finance the expensive, extensive way of farming corn. The question is whether the opportunities open to these young people actually increased due to this new second-generation ethanol value chain. The settled, older generation farmers who were targeted, may not have needed to extend their capabilities in the way the Poet/DSM plan was offering. In this case, there was a mismatch between the promise of new opportunities and the existing capability set of the farmers who might be included.

#### Investing in New Conversion Factors

The farmers who did get involved in the Poet/DSM plant had quite a good negotiating position. Negotiations between the farmers and the plant operators extended over several years and involved several re-alignments of the design of the value chain. We might say that the Iowa farmers were able to co-construct relevant conversion factors in relation to the ethanol production value chain. This was due to other conversion factors such as the level of education and access to resources. This example shows very nicely how relevant existing capabilities can be included in a biobased value chain.

For instance, a main point of discussion was how much corn stover should be left on the field as fertilizer and how much could be handed over to the ethanol plant. Poet/DSM provided them with support to make this estimate, while some farmers thought it wasn’t sufficient for maintaining soil health and argued for different norms. In the end, with further insights from experts at Iowa State University, they reached an agreement which may be considered a compromise. This norm for how much stover could be taken of the field should be considered a conversion factor, as it allows the farmer to protect soil health and to receive financial benefit from the left-over corn.

Additionally, there were debates about who should harvest the stover. Poet/DSM offered a customer contract to farmers, doing the stover harvest and delivery for them, for relatively little additional cost. However, many farmers preferred a grower’s contract, where they would harvest and deliver the stover themselves, because this would make them more independent. This also entailed acquiring supplemental and expensive agricultural implements. Ownership of technology should also be considered a conversion factor because it opens up many opportunities, especially economic ones, but also the capability of controlling one’s direct environment.

In the Iowa case it was relatively straightforward for farmers to invest in such conversion factors themselves, in collaboration with Poet/DSM because they could rely on other conversion factors. This was much less the case for farmers in South Africa and Jamaica.

Table 1 provides an overview of the different design strategies that we derived from the three cases and how they were implemented.

**Table 1:** Overview of different design strategies in each of the cases

	Jamaica	South Africa	Iowa
Design for existing conversion local factors	• Stay with sugar cane as the main crop • Choose well-known process technologies for ethanol • Apply advanced bagasse-to-energy technologies	• Offer tobacco- based alternative to tobacco farmers • Multi-purpose crop to allow choice for farmers	• Build on existing infrastructure for collection of biomass • Build on available high-tech skills by introducing high- tech processing technologies
Invest in new conversion factors		• Investment in active learning practices • Building a new local value chain	• Co-construction of new norm for how much stover can be left on the field • Ownership of new technologies by Iowa farmers
Adaptive design for new capabilities	• Leave open the possibility of using more advanced technologies in a later stage		

## Identifying Capabilities

What each of these cases shows is the importance of identifying for which capabilities a value chain is set up and for whom. If this isn’t done, the set-up of the value chain may not be inclusive and therefore may be less successful. As mentioned, if the value chain design is not aligned with local conversion factors, it may fail. Additionally, a specific value chain may not offer a new capability or sufficiently extend an existing capability for local biomass producers and be unattractive.

In the Iowa case, the corn farmers themselves weren’t necessarily looking to diversify their income. They already had this opportunity through the first-generation ethanol plant. Although the Poet/DSM plant extended their capability of diversifying their income, many of them choose not to. Similarly, the Solaris crop was designed to extend the capabilities of tobacco farmers to secure financial stability yet, as it turned out, they did not need to extend those capabilities.

This is a classic dilemma in designing for development. Oosterlaken et al. (2012) mention the case of ICT telecentres that were intended for specific wellbeing goals such as improved livelihoods but which were instead used for entertainment purposes (Ratan & Bailur, 2007). Creating opportunities for specific actors does not ensure that the actors will actually employ them. If developers of biobased value chains want to increase the likelihood that the opportunities they offer are actually seized by the intended beneficiaries, they may be wise to make sure these opportunities are indeed lacking in the specific context and deemed desirable by prospective biomass producers.

This may be especially challenging for biobased value chains as these often take shape within existing biomass producing practices. In developed countries, biomass producers may already have plenty of opportunities and not need new ones. But this may also be the case in the Global South – as we saw with the Solaris crop where the number of tobacco farmers looking for new opportunities turned out to be lower than expected.

### Capabilities for Participation

Oosterlaken et al. (2012) suggest that participation of local stakeholders is crucial in identifying which capabilities are in need of further development in a specific context. Other authors such as Alkire (2005) develop approaches for identifying capabilities that involve intense participatory processes with relevant communities. However, there are several issues with this approach, which Oosterlaken herself also mentions. Firstly, there is the matter of adaptive preferences, which Sen also initially identified (Sen, 1999). This implies that actors may adapt their expectations to the circumstances they are in. They may limit their hope for a better life if they are surviving in dire conditions. When asked about which capabilities they might still want to develop, their response is likely to be modest and may not include any new capabilities at all. Oosterlaken et al. (2012) remain optimistic about dealing with such adaptive preferences and believe that in the right setting, individuals will come up with a list of their preferred capabilities.

Secondly, not all participants will have the capability of participating in a constructive discussion about what their future should look like (Simpson & Basta, 2018). This may be due to institutional factors such as unreliable governance leading to low trust amongst individuals in that society (Postal et al., 2020), or cultural norms that prescribe that women shouldn’t publicly voice their opinion (Oosterlaken et al., 2012), or mobility issues due to bad roads or lack of transport.

### Differing Capabilities for Participation in The Cases

In the case-studies we describe, both barriers to participation were present. In the Jamaica and South Africa case-studies, many of the farmers we interviewed stated that their highest need was for higher income. More income would undoubtedly make their lives better, however, there may be many additional factors that are also needed for a truly better quality of life, such as better mobility or better education. This is actually one of the motivations behind the capability approach: to move beyond quantitative indicators such as income and instead to see what people are actually capable of achieving in a specific context. However, broader factors that could contribute to a better life were hardly ever brought forward by farmers in either South Africa or Jamaica, which may point to adaptive preferences because of low expectations. It could also indicate that to identify capabilities beyond financial security requires a more intricate form of interaction that requires both time and investment in mutual trust, not possible in the limited time available for these case-studies.

However, we also noticed that the capability of participating in decisions on the setting up of the value chains differed widely among the communities we observed. These barriers were mainly due to the social and cultural distance between actors setting up value chains and those providing the feedstock for it. These barriers to effective participation will likely also complicate the effort to identify which capabilities besides a higher income may be relevant to prospective partners in value chains.

For instance, the farmers in Iowa had good access to relevant scientific knowledge and were used to making decisions independently. They could negotiate directly and as equals with the other actors in the value chain, such as the operators of the ethanol plant, about how it should be set up. This was not the case for members of RBN in South Africa. RBN negotiated with Sunchem, but this was mostly done through the RBN management. While the actual farmers had input into these negotiations, they did not speak directly to Sunchem. For Jamaican sugar cane producers there was the issue of historical power structures, complicating direct interaction about decisions on the setting up of a value chain.

A possible solution to this might be the use of pre-defined capabilities, such as suggested by Nussbaum (2000). Sen was always against such an approach because he thought people should be able to define for themselves what the good life is (Robeyns, 2017), so relevant capabilities should always be determined through democratic processes. However, in the context of global biobased value chains, such democratic processes are too demanding for the actors setting up that value chain. Hence, a predefined list may be the best approach to this, although this list should be based on the specific context in which the value chain is to be located. A country- specific capabilities list such as provided by the UNDP development report, complemented with local observations, may be the best way to proceed. In cases where the social and cultural distance between the biomass producers and the value chain developers is not big, participatory approaches may be useful. The main point here is that to build a successful value chain, attention is needed to identify relevant existing and missing desirable capabilities.

## Conclusion

The question we posed as our main research question was how to achieve meaningful inclusion in global biobased value chains? We argued that the capability approach can be a useful framework for addressing this question and that it can guide the design of such value chains. Specifically, this involves three main strategies, namely designing for existing conversion factors, adapting design to emerging capabilities and investing in new conversion factors. These can be used to develop biobased value chains in alignment with the values, knowledge, skills and interests of local biomass producers. We have shown how these strategies may be applied in three distinct case-studies in Jamaica, South Africa and Iowa, USA. We thereby focussed on the capabilities of financial security and that of control over one’s environment which we framed as the capability of participating. Each of these capabilities materialized differently in the different case-studies and hence called for different choices in the design of the specific value chain. These differences underscore the need to identify *local* capabilities and conversion factors and adapt a prospective value chain accordingly, so as to increase both its moral legitimacy as well as its economic and sustainable effectiveness. This is especially relevant for biobased value chains as these are often built on top of existing practices. To be relevant to biomass producers, the new biobased value chains must offer some new kind of opportunity. However, this approach can also be useful for other agriculture-based value chains.

## Footnotes

1 The Global South refers to countries which are usually considered as ‘less developed’ in terms of institutions such as reliable goverance and in terms of infrastructure such as reliable energy provision, roads and healthcare, which is often due to colonialization and its continuing impact.

2 These cases are also described in Robaey et al. (2022), for the Jamaica and US case.

3 This data can be accessed at 4TU.ResearchData, DOI: 10.4121/19944404.

4 In some contexts, ethanol has been criticized for driving up food prices when derived from edible crops. The cases we describe in this paper have a different context. Ethanol is either derived from non-edible crops (in Iowa) or edible crops for which no demand exists (Jamaica).

## Notes

This paper is part of the Special Issue titled, “Just Food Production in a Changing Climate,” that has been edited by Hanna Schübel, Ivo Wallimann-Helmer, and Matthias Eggel
